# Preferential targeting of co-evolving Gag residues in long-term non progressors

**DOI:** 10.1186/1742-4690-9-S2-P278

**Published:** 2012-09-13

**Authors:** J Sunshine, K Shekhar, D Heckerman, AK Chakraborty, N Frahm

**Affiliations:** 1 University of Washington, Seattle, WA, USA; 2 Massachusetts Institute of Technology, Cambridge, MA, USA; 3 Microsoft Research, Los Angeles, CA, USA; 4 Fred Hutchinson Cancer Research Center, Seattle, WA, USA

## Background

A recent analysis of mutational patterns within Gag revealed independently evolving groups of residues (termed sectors) whose mutations are collectively coordinated. Of these sectors, sector 3 is the least tolerant of multiple simultaneous mutations and therefore is proposed to be the most vulnerable to a targeted immune attack. We hypothesized that coordinated CTL targeting of sector 3 residues is associated with immune control.

## Methods

We completed a comprehensive evaluation of Gag-specific responses in a cohort of 9 Long-term non-progressors (LTNPs, VL <2000 RNA copies/ml, untreated) and 9 HIV progressors (VL>10,000 RNA copies/ml, untreated). A Gag peptide set of 11-mer peptides overlapping by 10 amino acids was generated to reflect all variants found in at least 5% of clade B sequences in the LANL HIV Sequence Database. This peptide set includes 1300 peptides and covers all 500 amino acids of Gag. All study subjects were screened for responses to all peptides by IFN-γ/IL-2 FluoroSpot.

## Results

We observed a trend in the preferential targeting of sector 3 residues by LTNPs (p=0.07). This trend was not observed for any other sector or in total breadth of responses. Supporting the importance of sector 3 targeting, we found a significant positive correlation in our cohort between the relative proportion of sector 3 responses and CD4 count (r=0.49, p=0.04). We found no significant differences between LTNPs and HIV-Progressors in either the targeting of conserved 11-mers or overall Gag epitope variant recognition. Interestingly, LTNPs demonstrated higher levels of variant recognition than HIV-progressors when considering only the variable regions containing sector 3 residues.

## Conclusion

We found that preferential targeting of sector 3 residues distinguished Gag-specific responses between LTNPs and HIV-progressors, and that coordinated targeting of sector 3 residues may require cross-reactive responses. Additional investigations are ongoing to elucidate the role of sector 3 targeting in immune control of HIV.

